# The Effects of Shift Work on Sleeping Quality, Hypertension and Diabetes in Retired Workers

**DOI:** 10.1371/journal.pone.0071107

**Published:** 2013-08-16

**Authors:** Yanjun Guo, Yuewei Liu, Xiji Huang, Yi Rong, Meian He, Youjie Wang, Jing Yuan, Tangchun Wu, Weihong Chen

**Affiliations:** 1 Department of Occupational and Environmental Health, School of Public Health, Tongji Medical College, Huazhong University of Science and Technology, Wuhan, China; 2 Key Laboratory of Environment and Health in Ministry of Education & Ministry of Environmental Protection, and State Key Laboratory of Environmental Health (Incubating), School of Public Health, Tongji Medical College, Huazhong University of Science and Technology, Wuhan, China; Universität Bochum, Germany

## Abstract

**Background:**

Shift work has been associated with adverse health effects by disturbing circadian rhythms. However,its potential long-term health effects and the persistent effects after leaving shifts have not been well established.

**Methods and Results:**

We studied 26,463 workers from Tongji-Dongfeng Cohort in China. All the participants are retired employees of Dongfeng Motor Company. Information on demographics, occupational history and medical history were gathered through questionnaires. After adjusting potential confounders in the logistic regression models, shift work was associated with poor sleeping quality, diabetes and hypertension independently. We observed significant effects of shift work on poor sleeping quality, diabetes and hypertension; the ORs (95%CI) are 1.18 (1.09–1.27), 1.10 (1.03–1.17) and 1.05 (1.01–1.09) respectively. In the further analysis, we found elevated ORs (95%CI) for participants with poor sleeping quality, the ORs (95%CI) are 1.34 (1.08–1.60), 1.13 (1.05–1.21), 1.05 (1.03–1.07) and 1.05 (1.01–1.09) for 1–4, 5–9, 10–19, ≥20 years of shift work respectively. However, with the extension of leaving shift work duration, the effects of shift work on sleep quality gradually reduced.

**Conclusions:**

Shift work may be an independent risk factor for sleeping quality, diabetes and hypertension even in retired workers. Applicable intervention strategies are needed for prevention of sleep loss, diabetes, and hypertension for shift workers.

## Introduction

Shift work is an employment practice designed to make workers taking turns to have a rest and factories running all day. The term "shift work" normally includes both long-term night shifts and work schedules in which employees change or rotate shifts. With the rapid progress of industry worldwide, shift work itself remains necessary in many occupations and the demands of round-the-clock services have increased in some companies [1], [2]. Recent investigations reported that shift workers took up more than 20% of the whole working population in industry countries. And the population of shift workers is still on the rise in China [3], [4].

Our bodies are verified to have a regulatory mechanism to adjust the circadian rhythm [5]. Circadian rhythm mainly presents in sleeping and feeding patterns, and also in patterns of core body temperature, brain wave activity, hormone production and other biological activities. These patterns will be disturbed when circadian rhythms changes. Therefore, shift work is recognized as a risk factor for many health problems, and may cause many negative cognitive effects. Studies have confirmed that shift work could disrupt sleep patterns as well as physiologic circadian rhythms [6], [7], [8]. Recent studies have focused on the potential health effects of shift work on acute or chronic sleep deprivation, hypertension, cardiovascular diseases and series of metabolic disorders, such as type 2 diabetes and metabolic syndrome [2], [3], [9], [10], [11], [12].

Previous studies suggested that most shift workers were not able to adjust their circadian rhythms to the atypical timetable when they first started shift work [13], [14]. Some of them then suffer from sleep loss and poor quality of sleep. The results of Monk *et al* confirmed that shift workers suffered from worse sleep comparing with day workers, even in retirement [15]. In addition, the extension of shift work may cause more serious health problems. Recently, a long-term prospective study have reported that a modestly increased risk of type 2 diabetes was associated with an extended duration of shift work [2]. However, there are few studies focusing on the association between duration of shift work and the adverse health effects. Futhermore, it is still not clear wheather shift workers could get rid of the effects caused by shift work and reduce the health damage after leaving shifts position.

Therefore, we presented results from Dongfeng-Tongji cohort (DFTJ cohort) of 26463 retired workers. Our objectives were to quantify the association of shift work with sleeping quality, diabetes and hypertension, and to evaluate the persistent effects of shift work on sleeping quality after leaving from shift work.

## Methods

### Ethics Statement

The study protocol was approved by the institutional review boards of Dongfeng Motor Corporation (DMC) and Tongji Medical College Institutional review Board, School of Public Health, Tongji Medical College, Huazhong University of Science & Technology (Wuhan, Hubei, China). All participants provided written informed consent.

### Study Population

We used data from the baseline survey of DFTJ cohort which has reported in previous study [16]. In brief, 27,009 retired workers were included in the cohort if they were covered by Dongfeng Motor Corporation’s (DMC’s) health care service systems and agreed to provide baseline blood samples and questionnaire information between September, 2008 and June, 2010. Standard questionnaires and physical examinations were performed at baseline to collect and update information on demographics, occupational history, medications, self-reported medical history, and lifestyles. Information was collected by trained interviewers through face to face interviews. Trained investigators entered questionnaires into computer twice using EpiData software. And a group of trained investigators performed quality control by rechecking the entered data. In this study, participants were excluded if they provided no information on demographics (*n* = 204), occupational history (*n* = 241) or sleeping quality (*n* = 101). Finally, 26,463 participants were included.

### Ascertainment of Shift Work

Information on shift work was collected via questionnaires. Shift work was defined as any work schedule involving unusual or irregular working hours as opposed to a normal daytime work schedule: 8∶00 AM to 17∶00 PM. According to the distribution of shift work years, we categorized shift work into five categories: never, 1–4, 5–9, 10–19, ≥20 years in the final analysis.

Years after leaving shift work were defined as the duration from the year they left shift work to 2008. Years after leaving shift work was then grouped into four categories: <10, 10–19, and ≥20 basing on the grouping method of shift work in the final analysis.

### Ascertainment of Sleeping Quality

Sleeping quality in the last year was evaluated by three pre-specified response categories: normal quality, impaired quality, and poor quality. The questions were designed according to Pittsburgh Sleep Quality Index (PSQI). Normal quality was defined as (1) falling sleep in 30 minute, (2) hard to weak up at night, (3) having a high spirit in daytime. Poor sleeping quality was defined as having symptoms of sleep deprivation, such as insomnia, taking sleeping peels. And impaired sleeping quality was slightly impaired and represented the status between normal sleeping quality and poor quality; participants in this group had one of the following situations (1) difficulties in falling asleep (≥3 times per week); (2) easily wake up at night (≥3 times per night); (3) have night mares at night frequently (≥3 times per week).

### Ascertainment of Diabetes and Hypertension

The fast glucose was tested by ARCHITECT CI8200, Abbott, USA for participants. In our study, diabetes was diagnosed if participants met any of the following standards: (1) fasting plasma glucose level ≥7.0 mmol/l; repeated measurements were done to confirm the diagnosis on different days if the participants do not have any symptoms of diabetes. (2) self-reported clinically diagnosed diabetes (by glucose tolerance test) and using hypoglycemic drugs.

Individuals were defined as hypertension if they meet one of the following standards: (1) self-reported physician diagnosis hypertension; (2) systolic pressure ≥140 mmHg or diastolic pressure ≥90 mmHg. Each participants was diagnosed after determining three times of resting blood pressure in this study [16], [17], [18].

### Statistical Analyses

Demographic, lifestyle and occupational characteristics distributions at baseline were compared between rotating shift workers and day workers by Chi-square test and Student-T test for classified variables and continuous variables respectively.

Logistic regression model was used to calculate the odds ratio (OR) and 95% confidence interval (CI) for sleeping quality, diabetes and hypertension according to shift work and duration of shift work. The comparison group was retired workers who hadn’t engaged in shift work, In the multivariate-adjusted models, we adjusted for gender, age (<60 y, 60–75 y, ≥75 y), race (Han, others), marital status (single or divorced, married), current smoking status (no, yes), passive smoking (no, yes), current drinking status (no, yes), tea or coffee consumption (no, yes), life stress (no, yes), physical activity (no, yes) and body mass index (BMI) (<18.5, 18.5–24, 24–28, ≥28). For sleeping quality, we also adjusted chronic diseases (no, yes) including diabetes, chronic obstructive pulmonary diseases, all cancer and cardiovascular diseases. Because previous studies have reported that chronic diseases will have some impact on sleeping quality. Physical activities contain many aspects of activities, such as, walking, dancing, cycling, running, swimming and so on. Physical activity is defined as “yes” if the participant exercises ≥2 times per week and each time ≥20 minutes. Life stress is defined as ‘yes’ if the participants feel nervous, upset or even despair of daily life equal or more than 3 times per week. BMI was included in the multivariate-adjusted models, because studies have suggested that BMI was a mediate variable for diabetes. All *p*-values were two sided with a significant level at 0.05, and data were analyzed with SAS 9.1 (SAS Institute Inc. Cary, NC, USA).

## Results

A total of 26,463 participants (11,822 males, 44.7%) were included in this study. The average age of all participants was 63.6 years old in 2008. The baseline characteristics for participants are presented in Table 1. There are 9,118 (34.5%) participants engaged in shift work for at least 1 year. Percentages of current smoking, passive smoking and current drinking for shift workers were significantly higher than those of day workers (*P*<0.01). For education, there are 69.8% shift workers and 63.1% day workers attended junior high school or below. In shift workers, the percentage of BMI>24 was 52.4 which was lower than day workers (53.7%), but the difference is not statistically significant. Compared with day workers suffering poor sleeping quality (11.1%), shift workers with bad sleep (12.9%) was significant higher (*P*<0.01).

**Table 1:** The characteristics of the study population according to shift work.

Variables	Total	Shift work		*P* value
		Never	≥1 y	
**Gender**				<0.01*
Male	11,822	7,648	4,174	
Female	14,641	9,697	4,944	
**Age**				<0.01*
<60	7,745	4,618	3,127	
60–75	16,293	10,931	5,362	
≥75	2,425	1,796	629	
**Age (Mean±SD)**	63.589±7.796	64.216±7.801	62.396±7.647	<0.01#
**BMI**				0.21*
<18.5	1,374	910	464	
18.5–24	7,120	7,120	3,844	
24–28	6,844	3,264	3,580	
≥28	2,471	1,241	1,230	
**BMI (Mean±SD)**	23.887±5.154	23.938±5.136	23.788±5.187	0.28#
**Race**				
Han	26,702	17,099	8,973	0.27*
Others	391	246	145	
**Education**				<0.01*
Junior high school or below	17,310	10,942	6,368	
Senior high school or above	9,153	6,403	2,750	
**Marriage**				0.05*
Single or divorced	3,358	2,251	1,107	
Married	23,105	15,094	8,011	
**Current Smoking**				<0.01*
No	21,761	14,441	7,320	
Yes	4,702	2,904	1,798	
**Passive Smoking**				<0.01*
No	21,237	14,275	6,962	
Yes	5,226	3,070	2,156	
**Current Drinking**				<0.01*
No	20,965	13,921	7,044	
Yes	5,498	3,424	2,074	
**Physical activty**				0.04*
No	3,009	2,022	987	
Yes	23,454	15,323	8,131	
**Sleeping Quality**				<0.01*
Poor	3,096	1,922	1,174	
Impaired	14,209	9,417	4,792	
Normal	9,158	6,006	3,152	

Abbreviation: SD, standard deviation; BMI, body mass index.Variable are given as Mean±SD for numerical data, and frequency for categorical data.

* Chi-square test for categorical data;

# Independent sample t-test for numerical data.

In comparison to day workers, average blood pressure and fasting glucose for shift workers were significantly elevated (*P*<0.01) (Table 2). The levels of total cholesterol and high-density lipoprotein cholesterol for shift workers were lower than those of day workers. Hemoglobin and platelet count in shift workers were higher than day workers.

**Table 2:** The baseline levels of biochemical traits of the participants according to shift work (Mean±SD).

Variables	Total	Shift work		*P* value
		Never	≥1 y	
**Waist**	83.24±9.51	83.32±9.47	83.09±9.56	0.07
**Weight**	63.50±10.44	63.52±10.44	63.48±10.44	0.80
**SBP**	129.93±18.74	129.44±18.76	130.19±18.73	<0.01
**DBP**	77.88±10.89	77.58±10.86	78.47±10.94	<0.01
**Hematological** **traits**				
RBC (t/l)	4.56±0.47	4.56±0.46	4.57±0.48	0.19
WBC (g/l)	6.07±1.68	6.06±1.68	6.08±1.67	0.31
Haemoglobin(g/l)	136.58±14.42	136.15±14.21	137.37±14.77	<0.01
Platelet count(g/l)	186.90±56.86	184.93±55.67	190.59±58.86	<0.01
**Fasting glucose** **(mmol/l)**	6.07±1.74	6.01±1.73	6.10±1.75	<0.01
**Lipids**				
TC (mmol/l)	5.18±0.98	5.19±0.98	5.15±0.98	0.02
TG (mmol/l)	1.46±1.15	1.46±1.17	1.46±1.11	0.88
HDL-C (mmol/l)	1.44±0.41	1.44±0.40	1.43±0.43	0.03
LDL-C (mmol/l)	3.02±0.84	3.02±0.84	3.02±0.83	0.83

Abbreviation: SBP, Systolic blood pressure; DBP, diastolic blood pressure; RBC, red blood cell; WBC, white blood cell; TC, total cholesterol; TG, total triglycerides; HDL-C, high-density lipoprotein cholesterol; LDL-C, low-density lipoprotein cholesterol.

*P* values were calculated by independent sample t-test for numerical data.

**Figure 1:**
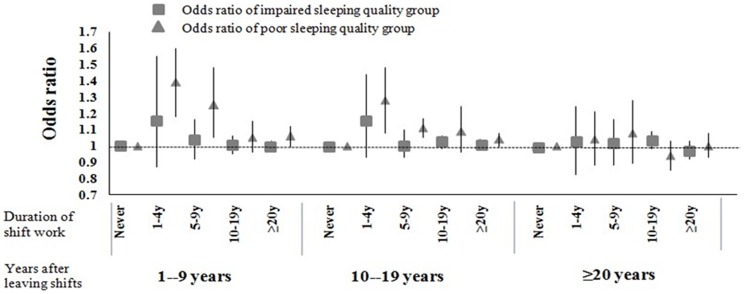
Odds ratio of impaired and poor sleeping quality according to duration of shift work and years after leaving shifts. The figure shows odds ratio of impaired and poor sleeping quality comparing to normal sleeping quality group according to duration shift work and duration of leaving shifts. The model adjusted for gender (male, female), age (<60 y, 60–75 y, ≥75 y), race (Han, others), marital status (single or divorced, married), tea consumption (no, yes), life stress (no, yes), current smoking status (no, yes), passive smoking (no, yes), current drinking status (no, yes), physical activity (no, yes), body mass index (<18.5, 18.5–24, 24–28, ≥28), chronic diseases (no, yes).

We examined the effects on sleeping quality, diabetes and hypertension according to the duration of shift work (Table 3). In both single factor logistic regression and multivariate-adjusted model, we observed slightly decreased relations of poor sleeping quality with the extension of shift work durations Compared with day workers, the ORs (95% CI) were 0.97 (0.93–1.01) and 1.18 (1.09–1.27) for shift workers with impaired sleeping quality and poor sleeping quality, respectively. In the categorical analysis, we found that the ORs of shift work on sleeping quality decreased when the duration of leaving from shift work extended (Figure 1). Shift work also increased ORs for diabetes and hypertension. Compared with people without shift work history, the ORs (95%CI) of shift workers are 1.10 (1.03–1.17) and 1.05 (1.01–1.09) for diabetes and hypertension, respectively. In the further analysis, we examined that persistently exposure to shift work would increase the ORs of diabetes and hypertension stably (Table 3).

**Table 3:** Odds ratio of all levels for sleeping quality, diabetes and hypertension by duration of shift work.

Variables	Duration of Shift Work				
	Never	1–4 y	5–9 y	10–19 y	≥20 y
**Number of participants**					
	17,345	1,562	1,334	3,205	3,017
**Sleeping Quality** *					
**Model 1**					
Impaired	1.00	1.16 (0.99–1.36)	1.06 (0.94–1.20)	0.99 (0.92–1.08)	0.94 (0.87–1.11)
Poor	1.00	1.36 (1.09–1.70)	1.32 (1.10–1.58)	1.24 (1.10–1.40)	1.02 (0.90–1.14)
**Multivariate-Adjusted Model**					
Impaired	1.00	1.19 (1.01–1.37)	1.01 (0.92–1.10)	1.00 (0.98–1.02)	0.99 (0.95–1.03)
Poor	1.00	1.34 (1.08–1.60)	1.13 (1.05–1.21)	1.05 (1.03–1.07)	1.05 (1.01–1.09)
**Diabetes**					
Model 1	1.00	0.92 (0.76–1.12)	1.01 (0.87–1.17)	1.10 (1.00–1.22)	1.16 (1.06–1.27)
Multivariate-Adjusted Model	1.00	0.99 (0.81–1.20)	1.06 (0.99–1.14)	1.07 (1.03–1.10)	1.05 (1.03–1.08)
**Hypertension**					
Model 1	1.00	0.97 (0.84–1.13)	1.02 (0.91–1.15)	1.06 (0.93–1.09)	1.04 (0.9–1.14)
Multivariate-Adjusted Model	1.00	1.03 (0.89–1.20)	1.05 (0.99–1.11)	1.03 (1.00–1.06)	1.02 (1.01–1.03)

Model 1 : single factor logistic regression.

Multivariate-adjusted model: adjusted for gender (male, female), age (<60 y, 60–75 y, ≥75 y), race (Han, others), marital status (single or divorced, married), tea consumption (no, yes), life stress (no, yes), current smoking status (no, yes), passive smoking (no, yes), current drinking status (no, yes), physical activity (no, yes) and body mass index (<18.5, 18.5–24, 24–28, ≥28).

* We adjusted chronic diseases (no, yes) except all the confounders in Multivariate-adjusted model.

The reference group was normal sleeping quality group and participants who did not work on shift position.

## Discussion

Our findings provided evidence that shift work was associated with increased risk of poor sleeping quality, diabetes and hypertension in retired workers. We found that short-term (<10 years) shift work mainly affected sleeping quality, but the impact on poor sleeping quality would last for 20 years after ceasing shift work. On the other hand, long-term (>10 years) shift work was associated with elevated risk of diabetes and hypertension. This study has significant importance for occupational health because of an increasing population on shift work worldwide.

The results of this study confirmed the elevated percentage of poor sleeping quality in shift workers, especially for those who involved in shift work for less than 20 years. Sleep is increasingly recognized as an important factor in the homeostasis of multiple body functions [19]. Many adverse health effects such as anxiety, endocrine disorders could hit the body following sleep deprivation [5], [14], [20], [21], [22]. Shift work, especially night rotating shift work, could reduce sleep quality directly by disturbing chronobiological rhythms [4], [23], [24], [25], [26] and reducing the secretion of melatonin [24]. During shift work, the workers have to change the bed time frequently [27], [28]. They would suffer from sleeping problems when the mismatch between their endogenous circadian rhythms and shift work appeared [29], [30]. Another reason to cause sleep deprivation was that some shift workers had to sleep at daytime which could be disturbed by bright light and noise. Similar results were reported by Gumenyuk, *et al.* They have discovered that lots of shift workers had problems in adjusting their circadian rhythms to the atypical timetable in the early duration of shifts [13]. Similarly, we observed that the influence on sleeping quality gradually decreased for those with long-term shift work of more than 20 years. Perhaps, after long-term shift work, the bodies may adapt to the rhythm of shifts slowly. Another possible reason is the selection by the participants of later chronotypes. The studies of Myriam Juda and colleagues suggested that chronotype modulated sleep duration and sleep quality in a population of workers employed in rotating schedules [31], [32]. The participants of later chronotypes experienced the highest constraints on sleep on morning-shift days and fewer effects were observed on their sleep quality. They would like to engage in long-term night shift work than others. Both selection by the participants of later chronotypes and adaptation may explain no significance effect on sleep quality of workers with longer night shift work duration.

Furthermore, our findings also suggested that sleep deprivation symptoms could be reduced after ceasing shift work. The effect on sleeping quality may return to the baseline level after 20 years of ceasing shift work. These results suggested that the first few years might be the most difficult time for shift workers to adjust themselves to regular circadian rhythms. More information or prevention on sleep deprivation should be provided to employees when they start shifts.

Our data suggested that ORs for diabetes significantly increased in participants working as shift workers for at least 10 years when compared with day workers. Several studies have reported that shift work was an independent risk factor for the onset of diabetes mellitus in Japanese population [1], [33], [34]. However, they didn’t consider the role of duration of shift work. We noted that risk of diabetes gradually elevated as the extension of shift work life in this study. The risk of diabetes was not increased for those engaged in shift work less than 5 years. The possible mechanisms inducing diabetes are shift work altered lifestyles and chronic work stress [35], [36]. The study conducted by Pan *et al* also revealed that shift work induced type2 diabets by increased BMI in American nurse’s cohorts [2]. But we didn’t observe the role of BMI in this study. In our study, average BMI for shift workers is a little lower than that for day workers. And percentage of BMI>24 in shift group (52.4%) is lower than day work group (53.7%), but there is no significant difference. Increased BMI in nurse’s cohort may attribute to eating habits of shift participants, and shift-workers need to be educated to cope better with shift-work related eating, sleeping and exercising habits [36].

The relationship between shift work and hypertension is still in debate. Several researches suggested that rotating night shift work was an independent risk factor for hypertension [7], [37], [38]. Possible mechanism of shift work inducing hypertension is that shift workers have a more activated sympathetic nervous system, greater endothelial dysfunction, more arterial stiffness, and different renal sodium handling. These factors increased the susceptibility to hypertension for shift workers [14], [37], [39]. Another study by Lieu *et al* also observed that black American were more susceptible to shift work related hypertension than white American because black American had a more activated sympathetic nervous system [37]. However, the study by Sfreddo *et al* indicated that night shift work did not increase blood pressure and was not associated with hypertension or pre-hypertension [40]. Our study of 9,118 shift participants and 17,345 day workers observed that the ORs of hypertension in shift group was significantly increased, especially in the participants engaged in shift work for more than 10 years.

The strengths of this study include its large sample size and detailed information on duration of shift work and other confounders. So far as we know, this is the largest study of retired workers to study shift work and its association with retired workers’ sleeping quality, and widely concerned aging people chronic diseases: diabetes and hypertension. Unfortunately, We cannot distinguish type 2 diabetes from type 1 diabetes in our study, but studies have confirmed that type 2 diabetes takes up 93% of diabetes patients in the elderly [41]. Furthermore, we did not collect data on dietary patterns, income and environmental noise; therefore we were unable to evaluate the confounding influences. However, the participants are from one big company and living in one town and diet patterns were likely to be relatively homogenous.

### Conclusion

In conclusion, this study suggested that shift work may be an independent risk factor for sleeping quality, diabetes and hypertension in retired workers. Shift work mainly affect sleeping quality in the early duration of shifts (<10 years), the effect on sleeping quality will decrease as time of leaving shifts extends, and the risk will come to the baseline after shift workers leave shift position for more than 20 years. The risks for diabetes or hypertension significantly increase after people worked on shifts for more than 20 years.
